# Brain structure correlates with auditory function in children diagnosed with auditory neuropathy spectrum disorder

**DOI:** 10.1002/brb3.2773

**Published:** 2022-10-03

**Authors:** Hannah E. Cooper, Lorna F. Halliday, Doris‐Eva Bamiou, Kshitij Mankad, Christopher A. Clark

**Affiliations:** ^1^ Developmental Imaging and Biophysics Section UCL Great Ormond Street Institute of Child Health London UK; ^2^ Faculty of Brain Sciences, UCL Ear Institute University College London London UK; ^3^ Audiology Department Royal Berkshire NHS Foundation Trust Reading UK; ^4^ MRC Cognition and Brain Sciences Unit University of Cambridge Cambridge UK; ^5^ National Institute of Health Research (NIHR) University College London Hospitals Biomedical Research Centre University College London London UK; ^6^ Department of Neuroradiology Great Ormond Street Hospital for Children London UK

**Keywords:** auditory neuropathy spectrum disorder, diffusion, hearing, MRI

## Abstract

**Introduction:**

Auditory neuropathy spectrum disorder (ANSD) is a term for a collection of test results which indicate disruption of the auditory signal at some point along the neural pathway. This results in a spectrum of functional outcomes, ranging from reasonably normal hearing to profound hearing loss. This study assessed brain structure changes and behavioral correlates in children diagnosed with ANSD.

**Methods:**

Seventeen children who had previously been diagnosed with ANSD were recruited to the study and underwent a battery of behavioral measures of hearing, language, and communication, along with structural MR imaging. Analysis of cortical thickness of temporal lobe structures was carried out using FreeSurfer. Tract‐based spatial statistics were performed on standard diffusion parameters of fractional anisotropy and diffusivity metrics. The control group comprised imaging data taken from a library of MRI scans from neurologically normal children. Control images were matched as closely as possible to the ANSD group for age and sex.

**Results:**

Reductions in right temporal lobe cortical thickness were observed in children with ANSD compared to controls. Increases in medial diffusivity in areas including the corpus callosum and in the right occipital white matter were also seen in the group with ANSD compared to controls. Speech perception abilities, both in quiet and in noise, were correlated with cortical thickness measurements for several temporal lobe structures in children with ANSD, and relationships were also seen between diffusion metrics and measures of auditory function.

**Conclusion:**

This study shows that children with ANSD have structural brain differences compared to healthy controls. It also demonstrates associations between brain structure and behavioral hearing abilities in children diagnosed with ANSD. These results show that there is a potential for structural imaging to be used as a biomarker in this population with the possibility of predicting functional hearing outcome.

## 1 INTRODUCTION

Auditory neuropathy spectrum disorder is a term for a collection of test results characterized by evidence of cochlear outer hair cell function (as shown by present otoacoustic emissions (OAEs) and/or cochlear microphonics (CM)) together with absent or grossly abnormal auditory brainstem response (ABR) indicating disruption of the signal at some point along the auditory neural pathway.

Pediatric cases of ANSD are generally detected shortly after birth via newborn hearing screening programs, with an estimated prevalence of around 10% of children with permanent hearing loss, which equates to around 1/10,000 of the general population (Feirn et al., 2013; Rance et al., 1999). The most significant risk factors are hyperbilirubinemia, hypoxia, infant respiratory distress syndrome, prematurity, low birth weight, intracranial hemorrhage, meningitis, sepsis, and gentamicin or vancomycin treatment (Beutner et al., 2007; Coenraad et al., 2011; Dowley et al., 2009; Morimoto et al., 2010). Up to 40% of cases of ANSD have a genetic origin including both syndromic and nonsyndromic causes (see Manchaiah et al., 2011, for review). Consequently, a number of sites of abnormal function have been identified along the auditory pathway, including presynaptic areas such as the inner hair cells and ribbon synapses, postsynaptic regions such as unmyelinated and myelinated auditory nerve dendrites and auditory ganglion cells, and auditory brainstem areas (Rance & Starr, 2015). This variation, while leading to similar test results on ABR and OAE/CM, leads to a spectrum of functional outcomes, which range from reasonably normal hearing with some minor difficulties hearing in background noise, through to profound hearing loss (Berlin et al., 2003).

It is particularly challenging to gain insight into neural processing in ANSD. ABR is unhelpful in evaluating the site of disfunction in cases of ANSD as it is, by definition, absent or grossly abnormal. Cortical auditory evoked potentials may give some information about degree of hearing, even where the ABR is absent or grossly abnormal (Gardner‐Berry et al., 2015). However, this technique is limited in its ability to assess central auditory structures and evaluate microstructural integrity and connectivity. Rance and Starr (2015) proposed using diffusion tensor imaging (DTI) to examine central auditory systems in ANSD in order to potentially identify site of lesion and evaluate changes in central auditory structures. A recent study of adults with X‐linked auditory neuropathy used fixel‐based analysis to evaluate fiber density of structures in the brainstem and showed reduced fiber density in both cranial nerve VIII and the auditory brainstem tracts (Zanin et al., 2020).

DTI has been used in several studies to look at relationships between white matter microstructure and functional auditory abilities in adults and children, with associations being observed between various speech discrimination and auditory processing tasks. Schmithorst et al. (2011) showed numerous brain areas with both positive and negative correlations between fractional anisotropy (FA) and speech‐in‐noise and filtered word scores in various white matter tracts bilaterally in normally hearing children aged 9–11 years, but did not examine mean diffusivity (MD) which may have given further insight into white matter microstructure by indicating areas of increased or decreased water diffusion. Atypical left ear advantage (LEA), sometimes used as a behavioral marker for auditory processing problems, has also been examined using DTI in children age 7–14 years with listening difficulties as reported by their parents. Lower FA values were observed in frontal white matter regions in the children with atypical LEA compared to controls (Farah et al., 2014). Correlations between temporal processing abilities, assessed using tone detection in noise measures, and diffusion metrics have also been evaluated in normally hearing adults and showed strong negative associations between tone detection in noise scores, and measures of diffusivity including MD and axial diffusivity (AD), particularly at the superior olivary complex (Wack et al., 2014). However, this study found higher diffusivity values with better signal detection levels suggesting decreasing white matter microstructural density with improving performance, an unexpected result. FA has been shown to be related to ABR latencies and wave intervals with increased FA at the inferior colliculus demonstrated in preterm or very‐low‐birth‐weight infants with shorter latencies for some waveforms. FA at the inferior colliculus was also positively correlated with wave V amplitude (Reiman et al., 2009).

It has also been suggested that diffusion metrics may be useful for estimating behavioral outcomes in children with hearing loss. Children with good outcome following cochlear implantation have higher FA values in brain areas associated with auditory and language function compared to those with poor outcome, with some authors suggesting that FA values may thus represent predictive biomarkers of cochlear implant outcome (Chang et al., 2012; Huang et al., 2015; Wu et al., 2014). Furthermore, a study of children with unilateral hearing loss evaluated using DTI suggested that better educational attainment was more likely with higher FA values in auditory regions (Rachakonda et al., 2014).

Cortical thickness measurements may also be useful in evaluating the impact of the distorted auditory input seen in ANSD on brain development, and there is evidence of reduced cortical volumes in the auditory regions in those with hearing loss independent of their language experience (Olulade et al., 2014).

There is a paucity of research evaluating the central consequences of the distorted auditory input experienced by children diagnosed with ANSD. The sensitivity of current diagnostic tools for ANSD is poor, giving little ability to evaluate disease processes and potential neural pathway alterations in this cohort. MRI methods including DTI and cortical thickness analysis may help to identify gross structural changes in neural anatomy and may lead to more targeted and effective management strategies for children with ANSD.

In this exploratory study, the hypotheses were as follows:

1. Children and adolescents diagnosed with ANSD show differences in cortical thickness in auditory areas compared to controls.
2. Children and adolescents diagnosed with ANSD show reductions in the density of white matter pathways as measured by diffusion parameters compared with age‐matched controls.
3. Brain structures (as measured by cortical thickness and diffusion metrics) correlate with clinical scores, including, pure tone audiometry, and speech detection in quiet and in noise in children and adolescents diagnosed with ANSD.

## 2 PARTICIPANTS AND METHODS

### 2.1 Group characteristics

Participants were recruited as part of a larger study, which entailed behavioral, electrophysiological and psychophysical measures of auditory function, as well as structural MR imaging. The study was approved by the Joint Research Ethics committee of GOSH/UCL Institute of Child Health and written informed consent was given by participants’ parents with assent/consent from the participants themselves as appropriate. A convenience sample of seventeen children with a diagnosis of ANSD were recruited from audiology clinics at several participant identification centers (PICs) throughout England. The study was also promoted via social media. PICs were asked to identify children age 6–16 years who had been diagnosed with ANSD on the basis of present cochlear function, demonstrated by OAEs and/or cochlear microphonic, and absent or grossly abnormal ABR bilaterally. Original evoked potential raw data was not available for all participants. Fourteen of the ANSD group were diagnosed through the UK newborn hearing screening (NHSP) care pathway for babies in the neonatal intensive care unit/special care baby unit. Twelve were premature (gestational age < 37 weeks); 13 required treatment for jaundice with eight receiving phototherapy and five receiving exchange transfusion. Two participants had no complications at birth but were referred for diagnostic audiology testing following no clear response on OAE screening. One participant was diagnosed at the age of 13 years following referral from their general practitioner due to difficulties hearing.

The control group comprised MRI data from neurologically normal children which was taken from the Developmental Imaging and Biophysics Section database. This database consists of MRI data from other studies for which consent has been given for data to be used in further investigations. Demographic information available for controls included age at scanning and sex. There was no explicit information about hearing or language abilities available for the control data. Control data was matched as closely as possible to ANSD participants for age and sex.

### 2.2 Behavioral measures

**FIGURE 1:**
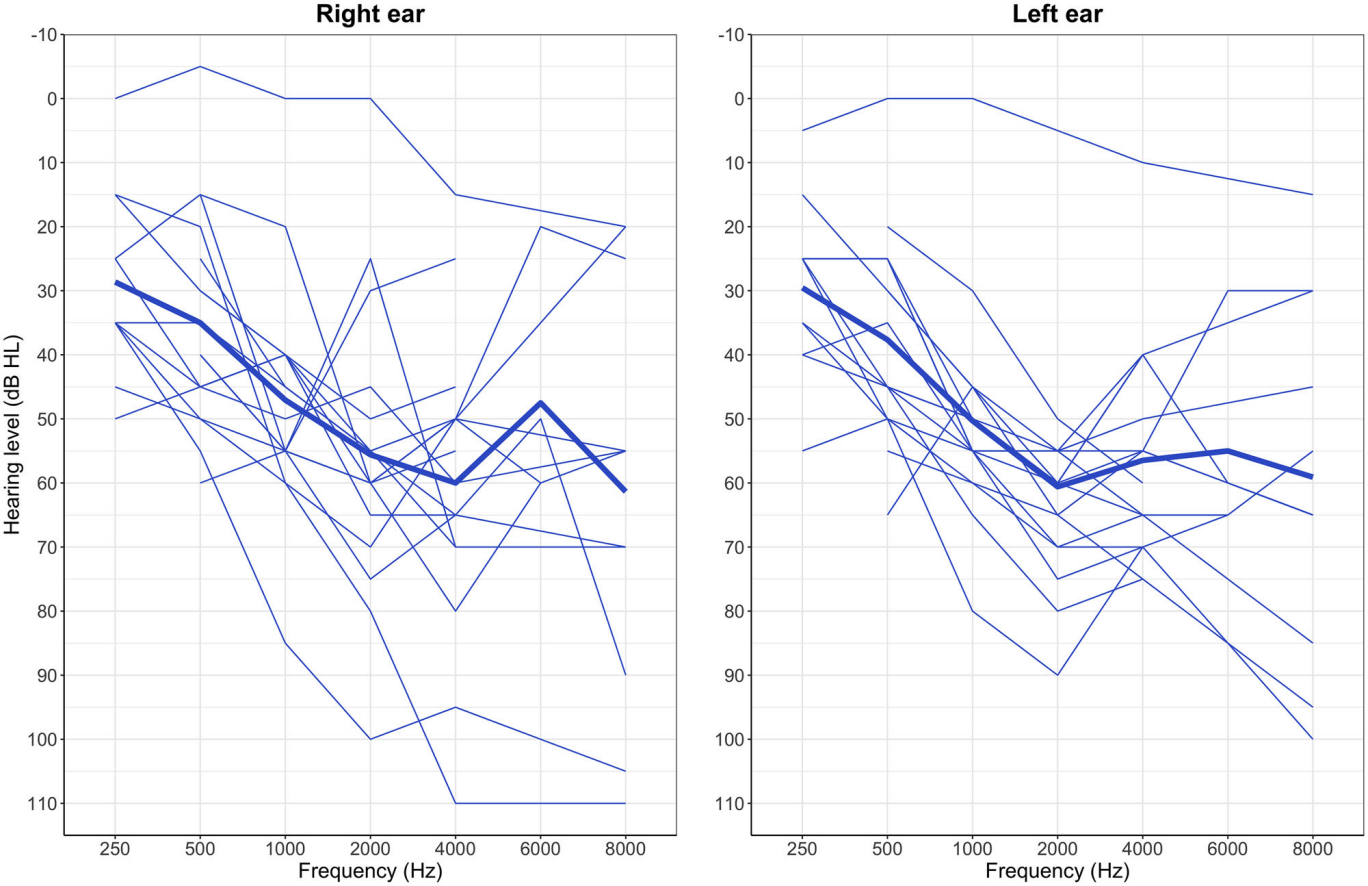
Speech‐frequency pure tone average thresholds as a function of frequency. Thin blue lines represent individual participant with thick blue lines representing mean values

Behavioral measures were conducted with the ANSD group only. Unaided pure tone audiograms (PTA) were acquired bilaterally for all children for at least four frequencies (0.5, 1, 2, and 4 kHz) bilaterally with results shown in Figure 1. Nonverbal IQ was assessed using the Block Design subtest of the Wechsler Abbreviated Scale of Intelligence (WASI; Wechsler, 1999), as IQ has been shown to be associated with diffusion metrics (Schmithorst et al., 2005), and is known to affect speech and language abilities (Rice, 2016). The Bamford‐Kowal‐Bench (BKB) sentence lists (Bench et al., 1979) were used to examine speech perception in quiet. The sentences were presented diotically through TDH39 headphones using recordings developed by UCL and the Medical Research Council Institute of Hearing Research (MRC/IHR) and were spoken by a British female speaker (Faulkner, 1998). Participants were asked to repeat what they heard in order to score each sentence. A modified version of the guidelines of the American Academy of Audiology (American Academy of Audiology, 2012) was used to calculate speech reception threshold. The starting presentation level was chosen to be above threshold based on interaction with participant and PTA results. If the participant was unable to comfortably identify the first three sentences the level was increased by 20 dB until the optimal level was reached. A bracketing procedure with step‐sizes of 20 dB down and 10 dB up was used until the 50% speech reception threshold (SRT) was determined.

**FIGURE 2:**
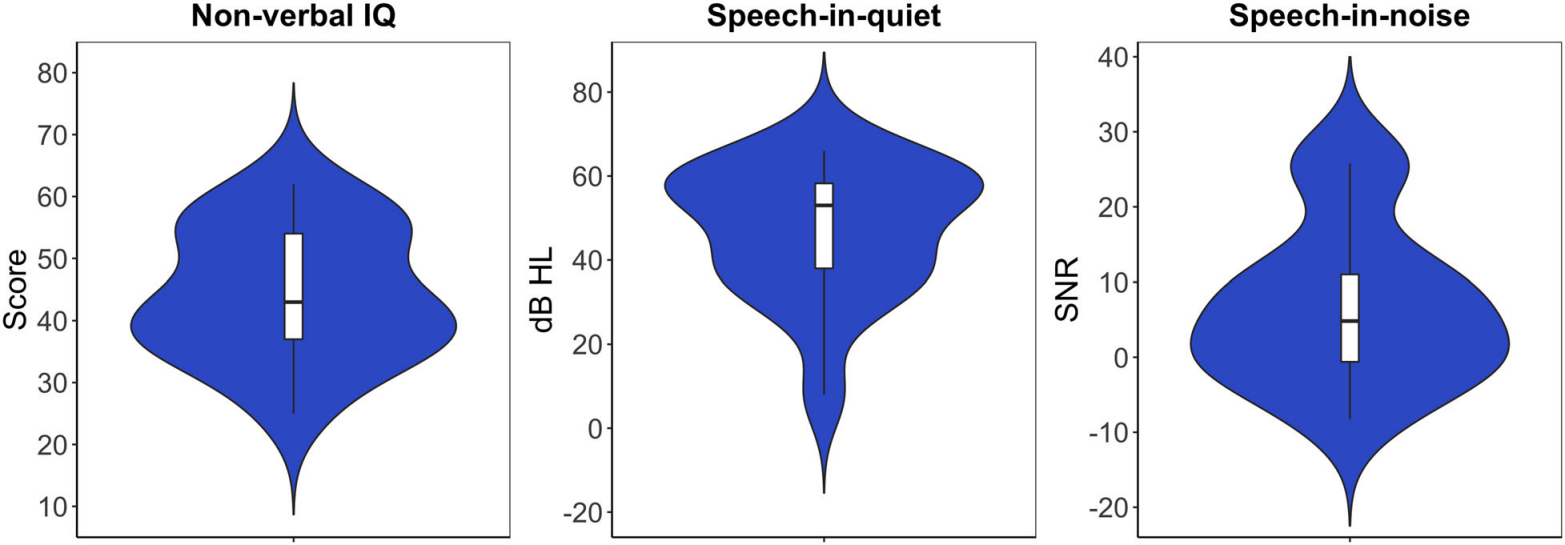
Performance on the nonverbal IQ, speech‐in‐quiet and speech‐in‐noise tasks. For nonverbal IQ, higher scores represent better performance. For speech‐in‐quiet (measured in dB HL) and speech‐in‐noise (measured in dB), higher scores represent poorer performance. The boxplot represents the 25th, 50th, and 75th percentiles for the group. The violin plot shows the kernel probability density, with the width of the plot representing the proportion of the data at that point

Speech‐in‐noise abilities were measured using the Children's Coordinate Response Measure (CCRM; Messaoud‐Galusi et al., 2011), an adaptive, nonstandardized test based on an adult version (Bolia et al., 2000; Brungart, 2001). During this test, participants heard a series of low‐context sentences using the carrier phrase “show the dog where the *[color] [number]* is” with colors being black, white, green, red, blue, or pink, and numbers being between one and nine, excluding bisyllabic seven. A speech‐shaped noise masker was used to add energetic masking. Participants were required to indicate what they heard via a response panel on a computer screen. Sentences were presented via Sennheiser HD25SPII headphones, with both speech and noise presented diotically. The output level of the test was kept constant at 70 dB SPL and a three‐up, one‐down adaptive procedure was used to vary the signal‐to‐noise ratio (SNR) and track 79.4% correct on the psychometric function (Levitt, 1971). The signal was audible to all participants, including those with more severe degrees of hearing loss. The task was carried out twice in succession and the mean of the two scores used in the analyses. A higher score indicated poorer ability to hear speech in the presence of background noise. Scores for nonverbal IQ, speech‐in‐quiet and speech‐in‐noise are shown in Figure 2.

### 2.3 MRI measures

MRI investigation was carried out on a 1.5‐Tesla Siemens Magnetom Avanto system (Siemens, Erlangen, Germany) and included T1‐weighted three‐dimensional fast low‐angle shot (FLASH) sequence (flip angle = 15°; TR = 11 ms; TE = 4.94 ms; voxel size = 1 mm isotropic; number of slices = 176); constructive interference in steady‐state (CISS) temporal bone sequence (flip angle = 70°; TR = 10.3 ms; TE = 5.1 ms; slice thickness = 0.70 mm; number of slices = 48); and diffusion tensor imaging consisting of a twice‐refocused spin echo diffusion‐weighted echo planar imaging (EPI) sequence with 60 unique gradient directions (*b* = 1000 s/mm^2^), interleaved with three images without diffusion weighting (*b* = 0 s/mm^2^) (TR = 7300 ms; TE = 81 ms; voxel size = 2.5 mm isotropic; number of slices = 60 axial). Control group MRI measures were carried out on the same system with the same parameters and software version. A consultant pediatric neuroradiologist (KM) evaluated all images from the ANSD group to determine the status of the internal auditory meatus and whether any gross abnormalities were present.

### 2.4 Region‐of‐interest analysis

Cortical reconstruction and volumetric segmentation of T1‐weighted images was carried out using FreeSurfer image analysis suite v5.2 for Mac OS X (Fischl, 2012). Technical details have been described in detail in the literature (Dale et al., 1999) therefore only a brief description is given here. Preliminary stages including motion correction and averaging, intensity normalization, automated Talairach transformation, and skull stripping were applied to the T1‐weighted volumetric images. The next steps included volume registration and removal of the neck, followed by segmentation of white matter and subcortical grey matter volumes. Inflation was then used to reveal topological defects in the segmentation. Neuroanatomical labels were applied to each location on the cortical surface parcellating the cortex into 68 regions of interest based on gyral and sulcal structure. The pial and white matter surfaces were reconstructed by FreeSurfer in order to estimate cortical thickness which was calculated as the closest distance between the grey/white matter boundary and the grey matter/CSF (cerebrospinal fluid) boundary at each vertex. All registrations and segmentations were checked visually to minimize methodological errors.

Analysis focused on cortical thickness of temporal lobe structures that contain the auditory cortex and areas involved in language processing including bilateral transverse temporal gyri, superior temporal gyri, middle temporal gyri and inferior temporal gyri. Total intracranial volume measures were also made for use in statistical analyses. Structures were tested for significant deviations from the normal distribution using the Shapiro–Wilk test. ANCOVA analysis was used to compare groups, controlling for age, sex and intracranial volume. For the ANSD group, partial Pearson's correlations were carried out between volumes of temporal lobe structures and speech discrimination measures (both in quiet and in noise), corrected for total intracranial volume.

### 2.5 DTI preprocessing

DTI data were visually inspected to check for the presence of motion artifacts. Volumes with artifacts present were removed. Data were preprocessed using TractoR version 2.6 (Clayden et al., 2011) and FMRIB Software Library (FSL) version 5.0 (Jenkinson et al., 2012). In brief, brain extraction was performed on a reference *b* = 0 volume for each subject (Smith, 2002). Diffusion‐weighted images were then registered to this reference volume to correct for eddy current distortions. At each voxel, a diffusion tensor was derived using a weighted linear least‐squares process to calculate voxel‐wise measurements of FA, MD, AD, and radial diffusivity (RD).

### 2.6 Tract‐based spatial statistics (TBSS) whole brain analysis

TBSS (Smith et al., 2006) was used to analyze the DTI data and was carried out using FSL version 5.0 (Jenkinson et al., 2012). Each subject's data were aligned to every other using nonlinear registration and the “most representative” image was identified as the target. This was achieved by registering each subject's data to every other subject, then summarizing every warp field by its mean displacement, and finally choosing the “most representative” subject as the one with the smallest mean distance to every other subject (Smith et al., 2006). All subject data were then transformed to MNI (Montreal Neurological Institute) space. A mean FA skeleton was created by aligning the FA images of all subjects to the most typical subject and thresholding (at FA = 0.2) to suppress areas of high intersubject variability or low mean FA. Each subject's aligned FA image was then projected onto the mean FA skeleton and voxel‐wise statistics were carried out on the skeleton FA data across subjects. Other diffusion parameters from the various models analyzed were projected onto the skeleton in a similar manner and these values used for voxel‐wise analysis. AD was defined as the first eigenvalue of the diffusion tensor and RD was calculated as the mean of the second and third eigenvalues. TBSS was carried out using nonparametric testing (5000 permutations). Threshold‐Free Cluster Enhancement was used as per the TBSS protocol to find clusters in data by comparing neighboring voxels to identify similarities, thereby increasing confidence that the results in each voxel have not occurred by chance. Family wise error (FWE) correction was used to reduce the likelihood of type I error. The locations of significant clusters were determined using FSL atlas tools (FSL, n.d). Age and sex were included as covariates in the analysis. Diffusion metrics have previously been shown to be affected by gestational age (see Pandit et al., 2013, for a review) and therefore this was also included as a covariate in the analysis.

### 2.7 Missing data

One participant completed behavioral testing but declined to be scanned. Scans were visually inspected for artifacts and one participant was excluded due to poor quality data caused by excessive head motion, leaving 15 T1‐weighted data sets from the ANSD group for analysis and 15 age and sex matched controls. Twelve of the 16 participants who completed T1‐weighted scanning went on to also complete diffusion weighted imaging. Scans were visually inspected for artifacts and two participants were excluded due to poor quality data caused by excessive head motion. This left 10 diffusion weighted data sets available from ANSD participants for DTI analysis and ten controls (age and sex matched where possible). Table 1 describes the demographics of the final groups. For region of interest (ROI) analyses, calculations were carried out both including and excluding participants whose scans were reported as abnormal by the neuroradiologist but the results did not substantially change for any evaluations. No participants with scans reported as abnormal by the neuroradiologist were included in the DTI analysis.

**TABLE 1:** Participant characteristics and between group comparisons

		ANSD		Controls					
Modality	Variable	*M*	*SD*	*M*	*SD*	Statistic (df)	*p*	Effect size	95% CI
T1 (*n* = 15 per group)	Age (years)	10.23	2.38	10.39	0.13	*W*(28) = 104.00	.740	0.48	[−2.06, 1.74]
	Sex	10 F:5 M	__	10 F:5 M	__	__	1.00	1.00	[0.20, 4.96]
DTI (*n* = 10 per group)	Age (years)	10.73	2.07	10.55	2.51	*t*(17) = 0.18	.863	0.08	[−1.98, 2.35]
	Sex	7 F:3 M	__	6 F:4 M	__	__	.999	1.52	[0.23, 10.86]

*Note*: Comparisons on age data were *t‐*tests or Mann–Whitney *U* tests. Group comparisons on sex were performed using Fisher's exact test. Effect size = Cohen's *d* for *t‐*test and Mann–Whitney *U* test, and odds ratio (OR) for Fisher's exact test. CI = confidence interval.

All participants were able to complete IQ and speech‐in‐quiet testing. However, five participants were unable to complete speech‐in‐noise testing, despite the signal being audible for all participants.

## 3 RESULTS

### 3.1 Radiological assessment

Sixteen participants diagnosed with ANSD completed T1‐weighted scanning. For these, abnormalities were reported in three, those being reduced brain volume overall (one participant), features of white matter disease of prematurity (one participant) and subtle cerebellar hypoplasia (one participant). Two of the participants with abnormalities had cerebral palsy. All participants had intact vestibulocochlear nerve bilaterally.

### 3.2 Region‐of‐interest analysis

#### 3.2.1 Group comparisons

Results of ANCOVA testing (controlling for age, sex, and total intracranial volume) showed significantly reduced cortical thickness of the right hemisphere in the ANSD group compared to controls (see Table 2). Reductions in cortical thickness in middle and superior right temporal lobe for the ANSD group compared to controls were also observed when controlling for age, sex, and total intracranial volume with strong effect sizes; however, these differences did not survive Bonferroni correction for multiple comparisons. No differences in cortical thickness were seen in the left temporal lobe.

**TABLE 2:** Group comparisons of temporal lobe and subcortical structures controlling for age, sex, and total intracranial volume

		ANSD (*n* = 15)		Controls (*n* = 15)					
Hemisphere	Structure	M	SD	M	SD	Statistic (df)	*p*	Effect size	95% CI
Left	Inferior temporal thickness (mm)	3.10	0.11	3.15	0.16	*F*(1) = 0.75	.393	−0.31	[−0.08, 0.14]
	Middle temporal thickness (mm)	3.11	0.26	3.22	0.21	*F*(1) = 3.04	.094	−0.62	[−0.02, 0.29]
	Superior temporal thickness (mm)	3.16	0.17	3.16	0.17	*F*(1) = 0.04	.836	0.00	[−0.11, 0.13]
	Transverse temporal thickness (mm)	3.04	0.36	3.21	0.26	*F*(1) = 2.85	.104	−0.65	[−0.04, 0.41]
	Mean thickness (mm)	3.08	0.09	3.11	0.05	*F*(1) = 2.61	.119	−0.57	[−0.01, 0.08]
Right	Inferior temporal thickness (mm)	3.22	0.32	3.27	0.11	*F*(1) = 0.74	.397	−0.30	[0.02, 0.28]
	Middle temporal thickness (mm)	3.15	0.37	3.35	0.15	*F*(1) = 5.57	.026	−0.86	[0.04, 0.44]
	Superior temporal thickness (mm)	3.18	0.24	3.30	0.14	*F*(1) = 5.26	.031	−0.76	[0.02, 0.28]
	Transverse temporal thickness (mm)	3.14	0.41	3.15	0.29	*F*(1) = 0.02	.892	−0.03	[−0.26, 0.30]
	Mean thickness (mm)	3.04	0.19	3.13	0.06	*F*(1) = 6.10	**.021**	−1.01	[0.02, 0.20]

*Note*: Comparisons that remained significant after controlling for multiple comparisons (Bonferroni; α = .0125 for individual thickness measurements, α = .025 for mean thickness measurements) are in boldface.

#### 3.2.2 Correlations with auditory testing

**FIGURE 3:**
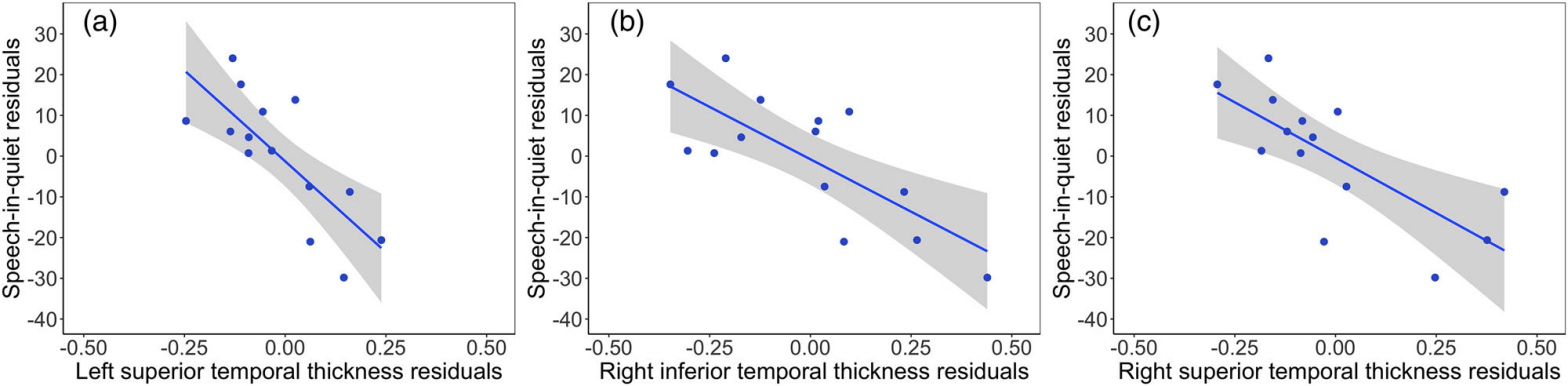
Partial Pearson's correlations are shown between speech‐in‐quiet scores and (a) left superior temporal thickness, (b) right inferior temporal thickness, and (c) right superior temporal thickness. Higher speech‐in‐quiet scores represent poorer performance. Age, sex, IQ, and total intracranial volume were included as covariates. The shaded panel represents standard error

**FIGURE 4:**
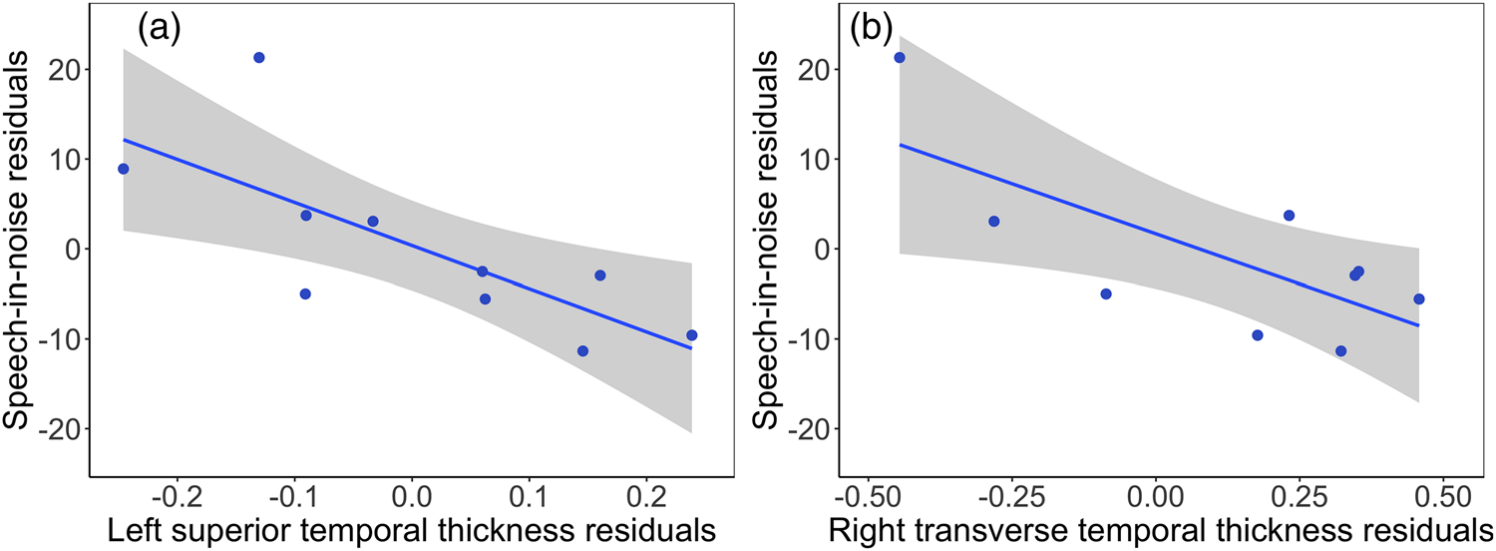
Partial Pearson's correlations are shown between speech‐in‐noise scores and (a) left superior temporal thickness and (b) right transverse temporal thickness. Higher speech‐in‐noise scores represent poorer performance. Age, sex, IQ, and total intracranial volume were included as covariates. The shaded panel represents standard error

Partial Pearson's correlations were performed between cortical thickness measures of the temporal lobes and both speech‐in‐quiet and speech‐in‐noise scores controlling for age, sex, IQ, and total intracranial volume for the ANSD group with results shown in Table 3. Significant correlations were seen for the speech‐in‐quiet scores for several temporal lobe structures including left superior temporal thickness (*r_p_*_ _= −0.78, *p =* .005) and right inferior temporal (*r_p_*_ _= −0.76, *p =* .007) and superior temporal (*r_p_*_ _= –0.70, *p =* .010) thicknesses, which all survived Bonferroni correction for multiple comparisons. Plots of significant relationships are shown in Figure 3. Significant correlations were also shown for speech‐in‐noise scores in the left superior temporal thickness (*r_p_*_ _= −0.78, *p =* .038) and the right transverse temporal lobe cortical thickness (*r_p_*_ _= −0.86, *p =* .010). The relationship with the right transverse temporal thickness survived correction for multiple comparisons. Plots of significant relationships are shown in Figure 4.

**TABLE 3:** Partial correlations between cortical thickness of temporal lobe structures and speech discrimination scores controlling for age, sex, IQ, and total intracranial volume

		Speech‐in‐quiet (dB HL)		Speech‐in‐noise (SNR)	
Hemisphere	Structure	Pearson's *r*	*p*	Pearson's *r*	*p*
Left	Inferior temporal thickness	−0.15	.652	−0.14	.764
	Middle temporal thickness	−0.36	.275	−0.23	.612
	Superior temporal thickness	−0.78	**.005**	−0.78	.038
	Transverse temporal thickness	−0.15	.655	−0.49	.264
Right	Inferior temporal thickness	−0.76	**.007**	−0.63	.127
	Middle temporal thickness	−0.58	.063	−0.42	.344
	Superior temporal thickness	−0.70	**.010**	−0.65	.111
	Transverse temporal thickness	−0.57	.066	−0.86	**.010**

*Note*: Comparisons that remained significant after controlling for multiple comparisons (Bonferroni; α = .0125) are in boldface.

### 3.3 Diffusion tensor imaging

#### 3.3.1 Group comparisons

**FIGURE 5:**
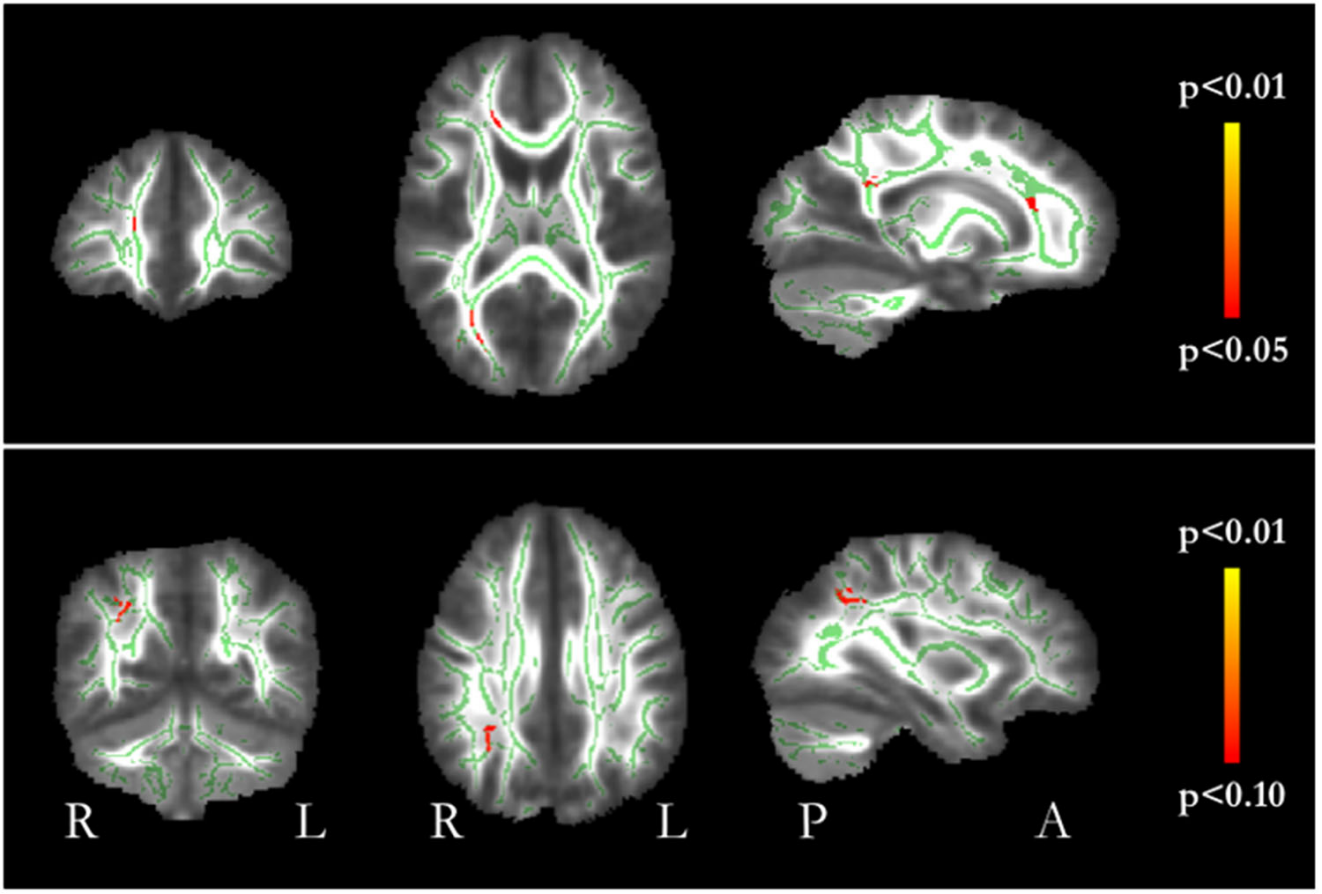
Whole brain group comparison TBSS results showing the cohort's mean which matter skeleton in green. Red voxels indicate areas where MD was significantly higher in ANSD participants compared to controls (*p* < .05, FWE corrected)

All ANSD participants who successfully completed DTI scanning had T1‐weighted scans reported as normal by the neuroradiologist. The results of TBSS analysis comparing ANSD participants and controls are shown in Figure 5. After controlling for the effects of age and sex there were no differences in FA, AD or RD. MD was significantly higher in the ANSD group (*p* < .05) in the corpus callosum and in the right occipital white matter.

#### 3.3.2 TBSS correlations with clinical scores

**FIGURE 6:**
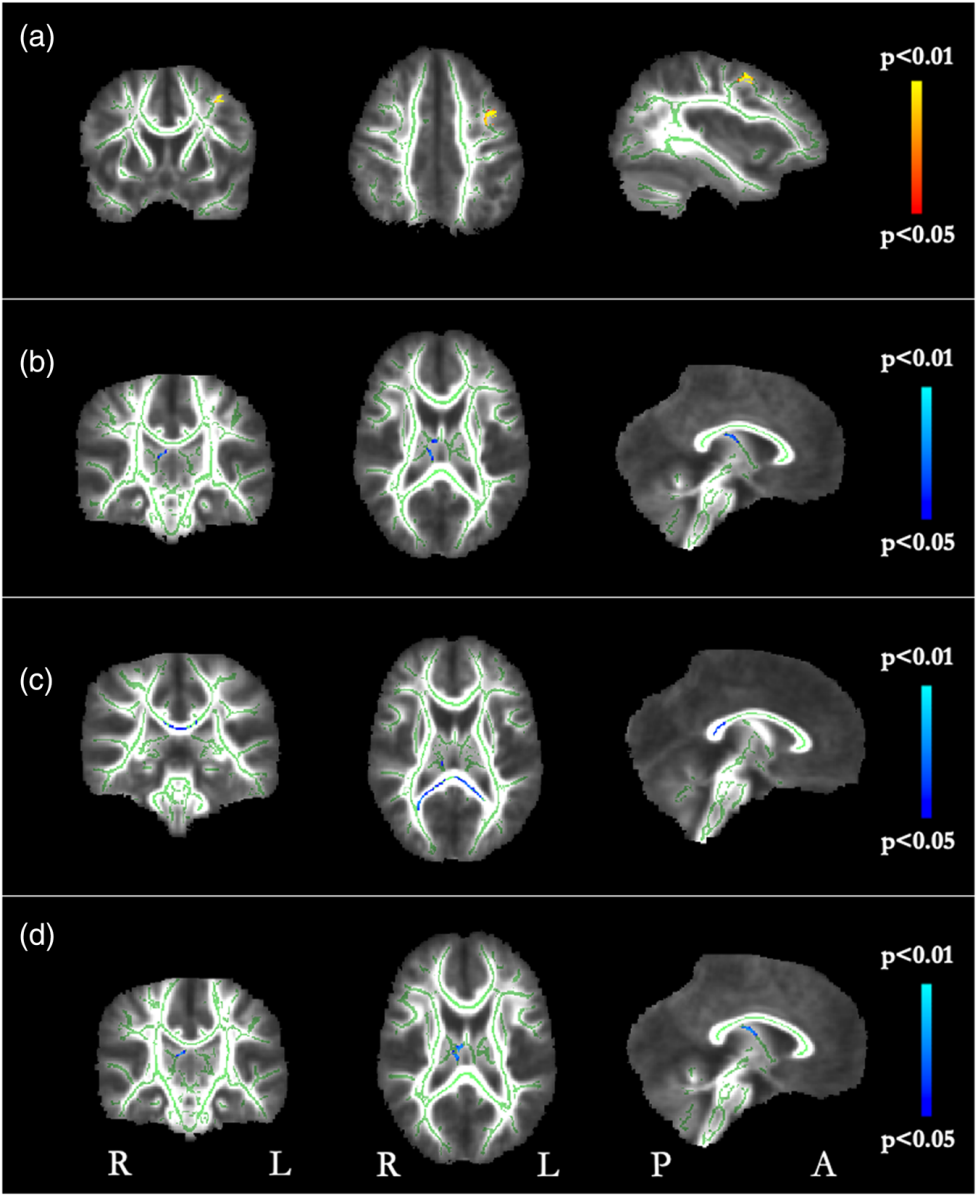
TBSS correlations with gestational age in weeks. (a) Red/yellow voxels show areas where gestational age and FA were significantly positively correlated (*p* < .05, FWE corrected). (b) Blue voxels show areas where gestational age and MD were significantly negatively correlated. (c) Blue voxels show areas where gestational age and RD were significantly negatively correlated. (d) Blue voxels show areas where gestational age and AD were significantly negatively correlated

Relationships between diffusion metrics and gestational age are shown in Figure 6. A strong positive relationship between FA and gestational age was seen in the left frontal lobe. There was a significant negative relationship between gestational age and MD in the right fornix/hippocampus. A significant negative relationship was seen between gestational age and RD in the splenium of the corpus callosum. There was a significant negative relationship with AD in the fornix. As significant relationships were found between gestational age and all diffusion metrics, gestational age was subsequently controlled for in the subsequent analyses.

**FIGURE 7:**
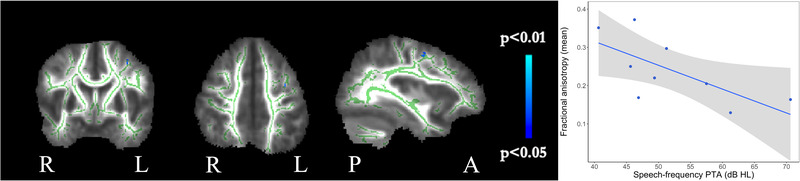
TBSS correlations with speech‐frequency pure tone audiometry (PTA). Blue voxels indicate areas where FA and PTA were significantly negatively correlated (*p* < .05, FWE corrected). Plot shows mean values of diffusion data from region‐of‐interest shown on the panels against speech‐frequency PTA

Results of TBSS correlations between white matter diffusion parameters and speech‐frequency PTA for the ANSD group only, and following the removal of one outlier (with speech‐frequency PTA that was substantially better than the rest of the group), are shown in Figure 7. After controlling for the effects of age, sex, IQ, and gestational age, a small volume with a significant negative relationship was found between speech‐frequency PTA and FA in the left frontal lobe. There was a significant positive relationship between speech‐frequency PTA and RD in the corpus callosum. Mean raw values of FA and RD from the ROIs with significant correlations were extracted and significant correlations were found (FA: *r_p_*_ _= 0.77, *p* < .001).

**FIGURE 8:**
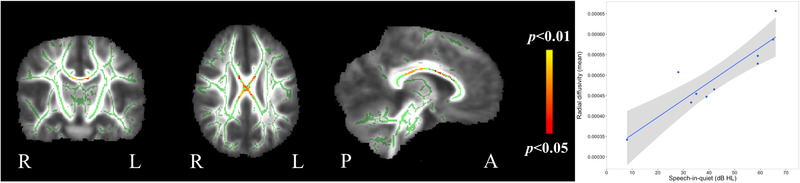
TBSS correlations with speech‐in‐quiet scores. Red/yellow voxels indicate areas where there was a positive correlation between speech‐in‐quiet scores and RD (*p* < .05, FWE corrected). Plot shows mean RD value from region‐of‐interest shown on the panels against speech‐in‐quiet

Results of TBSS correlations between white matter diffusion parameters and speech‐in‐quiet scores for the ANSD group are shown in Figure 8. After controlling for the effects of age, sex, IQ, and gestational age, a significant positive relationship was seen between speech‐in‐quiet scores and RD in the corpus callosum. Mean raw values of RD from the ROI with significant correlations were extracted and a highly significant correlation was found (*r_p_*_ _= 0.90, *p* < .001). No participants were identified as outliers in this analysis. There were no significant relationships seen for FA, MD, or AD.

## 4 DISCUSSION

The aims of this study were first, to examine differences in brain structure in children diagnosed with ANSD compared to controls, and second, to examine the relationships between brain structure and behavioral auditory function in children diagnosed with ANSD. We identified reductions in cortical thickness in the right temporal lobe as well as reduced white matter microstructural integrity in children with ANSD compared to controls. Increases in MD suggest a reduction in the density of white matter microstructure in children with ANSD compared to controls, particularly in areas including the corpus callosum and in the right occipital white matter. Moreover, relationships were observed between brain structure and auditory abilities.

### 4.1 Group comparisons

Mean total cortical thickness was reduced on the right in the ANSD group in this study. This is in common with a previous study which showed reduced mean whole brain cortical thickness but no significant reduction in grey matter volume in children and adolescents with sensorineural hearing loss (SNHL) compared to hearing controls (Li et al., 2012). ROI cortical thickness analysis showed differences in the right temporal lobe in children with ANSD compared to controls with significantly reduced thickness being observed in right middle and superior temporal gyri prior to correction for multiple comparisons. This is in contrast to the literature on SNHL which generally shows a preservation of cortical thickness or volume in the temporal lobes (Leporé et al., 2010; Shibata, 2007), as well as preserved leftward asymmetry of temporal lobe grey matter (Li et al., 2013; Shibata, 2007), although one study showed increased grey matter volume in the right superior temporal gyrus (Emmorey et al., 2003). However, all of these studies looked at participants with profound prelingual hearing loss who primarily communicated using signed language unlike the participants in the current study. The controls in previous studies were all oral language users and therefore used a different mode of communication to the deaf participants, introducing confounding factors into the studies. The results of the current study may suggest that different mechanisms contribute to cortical development in children with residual hearing who use spoken language to communicate, and in this case, with a diagnosis of ANSD, to those with profound hearing loss who communicate with signed language.

DTI analysis using TBSS showed significant differences in white matter structure in ANSD participants compared to controls with significantly increased MD seen in the mid body/isthmus of the CC and in the right occipital lobe as well as a trend towards increased RD in the region of the superior longitudinal fasciculus suggesting less dense white matter microstructure in these regions. A previous study of children with profound SNHL showed increases in white matter volume in the visual cortex, particularly on the right (Leporé et al., 2010), while other studies have shown decreases in white matter volume in the temporal lobes of profoundly deaf participants (Emmorey et al., 2003; Shibata, 2007) when manually drawing ROIs. The isthmus of the CC is thought to include connections from the superior temporal cortex and is therefore likely to be associated with language function (Witelson, 1989). Adolescents with profound hearing loss have also been shown to exhibit reductions in FA in areas including the splenium of the CC as well as bilateral areas of the temporal lobe on TBSS (Miao et al., 2012). The main differences between previous studies and the current study is that the children in this study all had usable residual hearing and all used spoken English as their primary mode of communication. Previous studies have concentrated on deaf individuals who use a signed language for communication. The results of the present study suggest that children diagnosed with ANSD who use spoken language have disrupted white matter microstructure in some brain areas.

### 4.2 Relationships between brain structure and auditory function

Negative relationships between speech discrimination scores and cortical thickness in left and right temporal lobe structures were observed in the ANSD group in this study, showing greater cortical thickness with better speech discrimination. Significant associations between speech‐in‐quiet scores and left superior temporal thickness as well as right inferior and superior temporal lobe thickness were observed following correction for multiple comparisons. There were also negative relationships between speech‐in‐noise scores and cortical thickness (equivalent to positive relationships between task performance and cortical thickness) in the left superior temporal gyrus and the right transverse temporal gyrus with the right transverse temporal gyrus relationship surviving correction for multiple comparisons. The left superior temporal gyrus contains the primary auditory cortex and is also an important cortical area for speech processing. Previous studies have shown that the right temporal lobe plays a crucial role in pitch perception particularly for complex stimuli (Zatorre, 1988) and has a stronger sensitivity to voices (Belin et al., 2000) than the left hemisphere. The ANSD group in the current study had significant difficulties with simple frequency discrimination tasks and with speech discrimination and this may have implications for the developing cortex. Also, the two speech discrimination tasks differed in several ways. The BKB sentences in quiet present an open‐set test in which the listener can use contextual cues to fill in any parts they may have missed. The CCRM, however, is a closed‐set test with low linguistic content. Therefore, the differences in locations of relationships with cortical thickness may reflect the different speech discrimination tests used.

In common with previous work (see Pandit et al., 2013, for a review), associations were seen between gestational age and diffusion metrics. Here, decreases in FA and increases in diffusivity measures were observed with decreasing gestational age, including in the left frontal lobe, fornix, hippocampus and corpus callosum. Prematurity is a well‐known risk factor for ANSD and 71% of the participants diagnosed with ANSD in this study were born before 37 weeks gestation. In order to try to limit the influence of gestational age on evaluation of relationships between diffusion metrics and auditory abilities, gestational age was controlled for in those analyses.

Previous studies have shown relationships between auditory processing abilities and FA in both normally hearing children and children with auditory processing difficulties. Schmithorst et al. (2013) suggested that atypical left ear advantage, which is used by some audiologists as a clinical indicator of auditory processing problems, is predicted by an increase in AD in the left internal capsule. A further study by the same group has shown decreases in FA and increases in MD in those children with left ear advantage compared to those with right ear advantage with areas of the frontal lobe and the corpus callosum being implicated (Farah et al., 2014). A previous study of children with sensory processing disorders showed associations between FA and auditory profile score as assessed by parental questionnaire with significant clusters located in the left and right posterior thalamic radiations and in the corpus callosum (Owen et al., 2013). In the current study, significant relationships between diffusion measures and auditory behavioral scores were observed in the ANSD group. Decreasing FA was seen with increasing speech‐frequency PTA suggesting a decreasing coherence in white matter microstructure with poorer hearing in the left frontal lobe and the corpus callosum.

Positive correlations between FA and various speech discrimination tasks have been shown in children in the absence of hearing problems, with significant areas being the corpus callosum, prefrontal cortex, and occipotemporal white matter. Negative correlations of FA with task performance have also been demonstrated, particularly in the posterior centrum semiovale (Schmithorst et al., 2011). A positive relationship between RD and speech‐in‐quiet scores was seen in the mid body/isthmus of the corpus callosum in this study, again suggesting decreasing white matter density in an area associated with language processing with poorer speech discrimination abilities.

### 4.3 Methodological considerations

Several limitations should be considered when interpreting our results. The first is that there was limited information about the control group particularly in relation to gestational age and IQ which would have been useful in order to control for these measures in the analyses. Information about the control group's auditory status would also have been helpful for ensuring that no hearing deficits were present. Data was unavailable as control data was drawn from a database of previously collected information rather than prospectively acquired. Second, the sample size for the ANSD group was small and, although there was some variation within the group, the majority of children had a moderate hearing loss with fewer at the ends of the spectrum. The study design meant that children with severe‐profound ANSD would necessarily be excluded as many will have cochlear implants and would therefore be unable to take part in MRI scanning. Third, several of the children in the ANSD group were born prematurely and several had other co morbidities apart from ANSD, including cerebral palsy and visual impairment, which may also be associated with structural brain changes. However, this would be expected in a typical cohort of children with ANSD and therefore may be more clinically representative of the population (Ching et al., 2013). Fourthly, although ABR testing was attempted on all participants with ANSD it proved challenging in many cases due to movement and issues with compliance. However, evidence of neuromaturation was seen in several participants who had visible waveforms on ABR testing. This may not be uncommon in this population with reports of maturation on ABR testing ranging from 7% to 85% (Attias & Raveh, 2007; Berlin et al., 2003); Uus, 2011). Fifth, over half (76%) of the ANSD group required treatment for jaundice as neonates and high levels of unconjugated bilirubin may have an impact on brain development. Very little information about bilirubin levels was available for the participants with ANSD (all was collected from parents) and none was available for controls. As this is a significant risk factor for ANSD, more detailed information should be collected and used in any future analysis (Wisnowski et al., 2014). Finally, there are known limitations of the DTI method, which may result in errors where there are crossing or kissing fibers, or partial voluming with CSF (Farquharson et al., 2013).

## 5 CONCLUSION

To our knowledge, this is the first study to show brain structural differences in children diagnosed with ANSD compared to healthy controls. It is also the first study to show associations between brain structure and behavioral auditory scores in children diagnosed with ANSD. These results suggest that the auditory difficulties experienced by children diagnosed with ANSD are related to brain structural abnormalities and that there may be the potential for structural imaging to be used as a biomarker for stratifying treatment options in this population, including prediction of auditory functioning such as speech perception outcomes.

### CONFLICT OF INTEREST

All authors declare that they have no conflicts of interest.

### PEER REVIEW

The peer review history for this article is available at: https://publons.com/publon/10.1002/brb3.2773.

## Data Availability

The data that support the findings of this study are available from the corresponding author upon reasonable request.
